# Real‐World Treatment of Patients With Sinus Node Dysfunction and Heart Failure With Preserved Ejection Fraction

**DOI:** 10.1111/pace.70172

**Published:** 2026-02-26

**Authors:** Nicole Habel, Sarah Rosemas, Lucas Higuera, Patrick Zimmerman, Markus Meyer

**Affiliations:** ^1^ University of Vermont Medical Center Burlington Vermont USA; ^2^ Medtronic, Cardiac Rhythm Management Mounds View Minnesota USA; ^3^ University of MN Dept of Medicine, Cardiovascular Division Minneapolis Minnesota USA

**Keywords:** heart rate, HFpEF, pacemaker, pacing

## Abstract

**Background:**

Permanent pacemaker (PPM) implantation is recommended in patients with sinus node dysfunction (SND). In patients with coexisting heart failure with preserved ejection fraction (HFpEF), clinicians may be hesitant to treat SND due to concerns about exacerbating heart failure. Little is known about real‐world PPM utilization and disease burden in the HFpEF population that has been assumed to benefit from low heart rates. This study aims to characterize the disease burden and PPM utilization in patients with SND+HFpEF versus SND‐only in a Medicare population.

**Methods:**

Patients diagnosed with SND were identified in the Medicare administrative claims database and stratified by presence or abscence of prior evidence of HFpEF. Rates of the following clinical outcomes were compared between the cohorts during follow‐up: PPM implant, HF hospitalization, atrial fibrillation (AF) hospitalization, second/third‐degree AV block or HFrEF, myocardial infarction (MI), ischemic stroke, and all‐cause mortality.

**Results:**

SND+HFpEF (*N *= 26,986) patients were older (81.1 vs. 78.5, *p* < 0.0001) and more likely to be female (63.5% vs. 49.9%, *p* < 0.0001) than SND‐only patients (*N* = 74,269). Median follow‐up time was 884 days (IQR 476–1408). The PPM implant rate during follow‐up was lower in the SND+HFpEF population (HR = 0.811, *p* < 0.0001). The SND+HFpEF population had higher HF and AF hospitalizations (HR = 2.229 and HR = 1.174, both p < 0.0001), and higher incidence of second/third‐degree AV block and HFrEF (HR = 1.364 and HR = 1.640, both *p* < 0.0001) than the SND‐only population.

**Conclusions:**

PPM implant rates were lower, and overall disease burden was greater in HFpEF+SND patients compared to SND‐only patients. Future work should focus on optimizing treatment pathways for patients with HFpEF and SND.

## 1 Introduction

Permanent pacemaker (PPM) implantation in patients with sinus node dysfunction (SND) is recommended to improve health status and quality of life. Patients with heart failure with preserved ejection fraction (HFpEF) tend to have many comorbidities, including SND, but the optimal treatment approach for HFpEF with SND is uncertain. Until recently, heart failure (HF) treatment guidelines suggested that patients with HFpEF may benefit from low heart rates by prolonging the diastolic filling period [1]. As a result, clinicians may be reluctant to implement pacing therapies in SND patients with HFpEF due to a perception that this may worsen their HF. However, little is known about real‐world PPM utilization and disease burden in this comorbid population. The objective of this study is to characterize the disease burden and PPM implantation rates in patients with comorbid HFpEF+SND compared to those with SND‐only in the Medicare fee‐for‐service (FFS) population, representing the US population ≥65 years.

## 2 Methods

### 2.1 Data Source

This retrospective observational database study uses the US Medicare 100% FFS 2016–2022 claims (inpatient, outpatient, and professional services claims, and enrollment data). The Medicare FFS database includes de‐identified, patient‐level information on the healthcare services covered for beneficiaries enrolled in Medicare Parts A and B. Utilization for individual beneficiaries can be linked over time and across providers. Detailed information submitted by providers from claims data includes but is not limited to, the following: an encrypted beneficiary identifier and beneficiary responsibility; provider identity; Medicare program payments; from and through dates; admission and discharge dates; information on source of admission and discharge destination (including death) for institutional providers; International Classification of Diseases Tenth Revision (ICD‐10) diagnosis and procedure codes; revenue centers, HCPCS/CPT (Healthcare Common Procedure Coding System/Current Procedural Terminology) codes, and charges associated with those services; and annual demographic and enrollment information for all Medicare beneficiaries. Sample selection and statistical analyses were undertaken with SAS software version 9.4 (SAS Institute Inc., Cary, NC, USA).

### 2.2 Sample Selection

Patients were eligible for the analysis if they had a diagnosis code for SND (ICD‐10 diagnosis code I49.5) between 2017 and 2021, and evidence of symptomatic bradycardia prior to or concurrent with the SND diagnosis (R00.1, R53.81, R53.83, R42, or R55) to increase the sensitivity of the SND diagnosis; the index date was assigned as the last date of the index encounter in which SND was diagnosed. Patients were required to have at least 12 months of continuous enrollment in Medicare Part A and B before the index encounter, no evidence of a cardiac implantable electronic device (CIED) during baseline or evidence of a first‐event CIED replacement in the follow‐up, and no systolic or combined HF (I50.2x or I50.4x) or second/third degree AV Block (I44.1, I44.2, I44.30, or I44.39) during baseline through the index encounter. Patients were stratified into two cohorts based on whether they had either one inpatient or two outpatient diastolic HF diagnosis codes (I50.3x) at least 30 days apart at any point before or during the index encounter. The presence of diastolic HF and the absence of systolic or combined HF and second/third degree AV Block, combined with the SND diagnosis, defines the HFpEF+SND population; the SND‐only population is defined by the SND diagnosis described before and the absence of diastolic HF, systolic or combined HF, and second/third degree AV Block. The baseline period consisted of the 12 months before the index date, and the follow‐up period extended from the index date to the end of continuous enrollment, end of database, or death, whichever came first.

### 2.3 Statistics

Patient demographics and comorbidities documented during the baseline period were summarized for patients with HFpEF+SND and those with SND‐only (please see Appendix Tables A1 and A2 for diagnosis and procedure codes used). Continuous variables were reported as mean and standard deviation; categorical variables were reported as frequency and percentage of patients.

Cumulative incidence rates of each clinical outcome during the follow‐up period were summarized for patients with comorbid HFpEF+SND and patients with SND‐only, including PPM implant, all‐cause mortality, HF hospitalization, AF hospitalization, ischemic stroke/TIA, myocardial infarction (MI), and incident diagnosis of high‐degree (second or third degree) AV Block, and incident diagnosis of Systolic/Combined HF (diagnosis and procedure codes listed in Appendix Table A1). Clinical outcomes were assessed as time‐to‐event outcomes using the Kaplan–Meier method. For all time‐to‐event measures, patients were censored upon disenrollment from Medicare, end of the database, or death; for the second or higher degree AV block endpoint, PPM implant was an additional censoring event.

Statistical comparisons of baseline characteristics between patient cohort groups (comorbid HFpEF+SND versus SND‐only) were conducted using Pearson's chi‐square tests for categorical variables and *t*‐tests for continuous variables. Fine‐Gray competing risks regression models were used to assess unadjusted and adjusted time‐to‐event comparative outcomes during follow‐up between patients with HFpEF+SND and patients with SND‐only. Adjustment variables included age, sex, geographic region, race/ethnicity, Charlson Comorbidity Index (a weighted index to predict the risk of death within 1 year of hospitalization for patients with comorbidities) [2, 3], and history of diabetes, hypertension, atrial fibrillation (AF), atrial flutter, chronic obstructive pulmonary disease (COPD), MI, obesity, chronic kidney disease (CKD) ≥stage 3, end‐stage renal disease (ESRD), cardiomegaly, pleural effusion, ischemic stroke, palpitations, secondary pulmonary arterial hypertension (PAH), angina, left bundle branch block (LBBB), tachycardia, and cardiac ablation.

## 3 Results

### 3.1 Study Cohort

**FIGURE 1:**
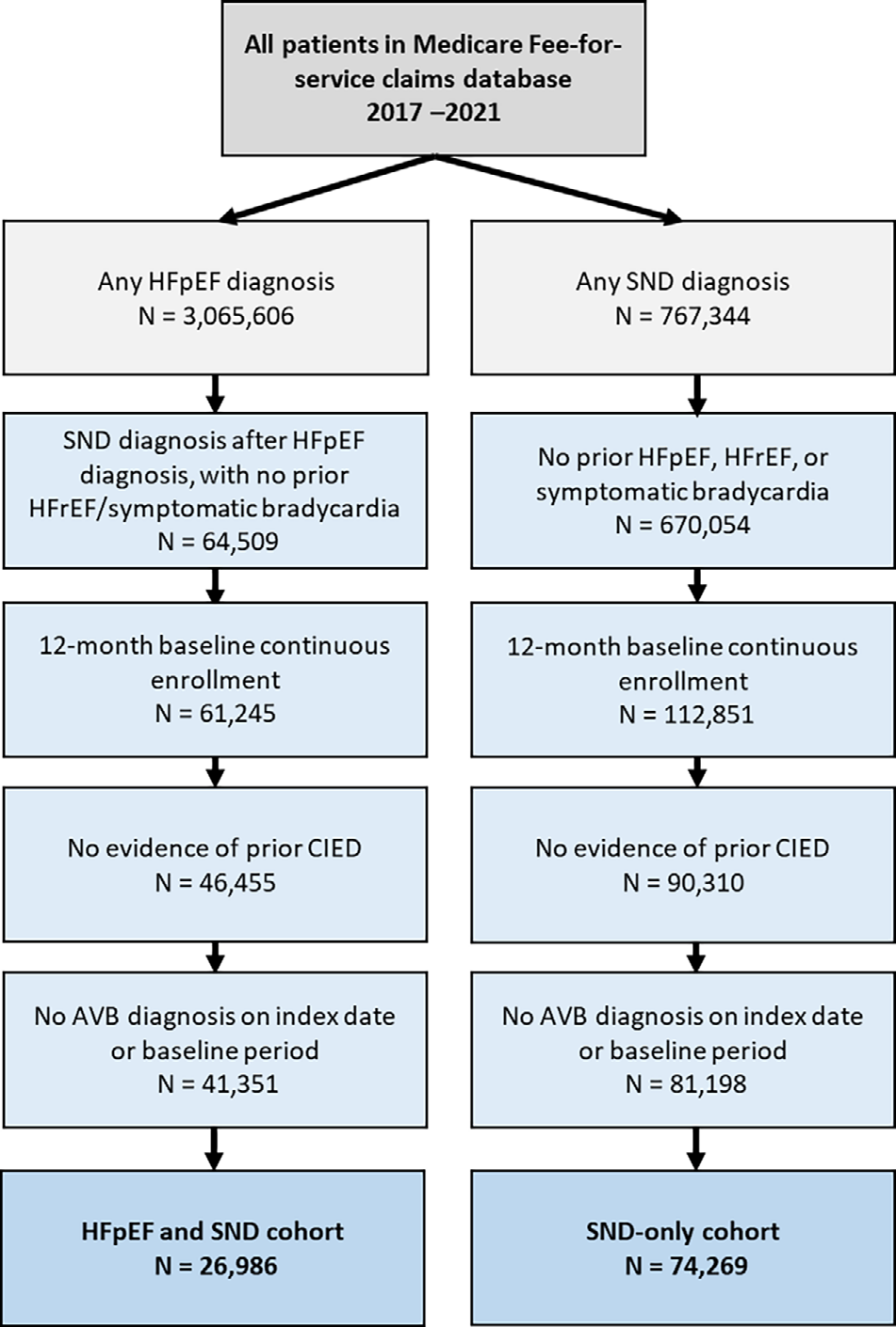
Patient selection flowchart. AVB, atrioventricular block; CIED, cardiovascular implantable electronic device; HFpEF, heart failure with preserved ejection fraction; HFrEF, heart failure with reduced ejection fraction; SND, sinus node dysfunction.

A total of 101,255 patients were identified. Of the total 26,986 (26.7%) had HFpEF+SND and 74,269 (73.3%) had SND‐only (Figure 1), with a follow‐up of mean ± SD of 708 ± 528 and 1038 ± 589 days, respectively (overall median follow‐up of 884 days, IQR 476–1408 days). Among HFpEF+SND patients, the majority (84.4%) were diagnosed with HFpEF prior to SND, with 479 ± 423 days between diagnoses, whereas 15.6% received a HFpEF diagnosis during the index SND encounter. Demographics and patient characteristics captured during the 12‐month baseline period are shown for each cohort in Table 1. Patients with HFpEF+SND were older (81.1 ± 8.8 years vs. 78.5 ± 9.0 years, *p* < 0.001), more likely to be female (63.5% vs. 49.9%, *p* < 0.001), and had higher Charlson index scores (4.3 ± 3.0 vs. 1.4 ± 2.2, *p* < 0.001) at baseline.

**TABLE 1:** Patient baseline characteristics in comorbid HFpEF+SND and SND‐only cohorts.

	HFpEF+SND	SND only	*p*‐value	SMD
***N***	26,986	74,269		
**Age, mean (SD)**	81.1 (8.8)	78.5 (9.0)	<0.0001	0.295
**Female, *n* (%)**	17,136 (63.5)	37,060 (49.9)	<0.0001	0.279
**Race/ethnicity, *n* (%)**			<0.0001	
Asian	459 (1.7)	1040 (1.4)		0.022
Black	2105 (7.8)	3788 (5.1)		0.111
Hispanic	324 (1.2)	817 (1.1)		0.016
N. Am. Native	135 (0.5)	371 (0.5)		0.004
Other	324 (1.2)	965 (1.3)		0.010
Unknown	124 (0.5)	691 (0.9)		0.056
White	23,532 (87.2)	66,694 (89.8)		0.080
**Charlson Index, mean (SD)**	4.3 (3.0)	1.4 (2.2)	<0.0001	1.104
**Baseline comorbidities, *n* (%)**				
Diabetes	11,091 (41.1)	17,602 (23.7)	<0.0001	0.378
Hypertension	24,098 (89.3)	52,211 (70.3)	<0.0001	0.489
Atrial fibrillation	18,431 (68.3)	28,445 (38.3)	<0.0001	0.630
Atrial flutter	3319 (12.3)	4501 (6.1)	<0.0001	0.216
History of ablation	521 (1.9)	1114 (1.5)	<0.0001	0.036
COPD	8474 (31.4)	9135 (12.3)	<0.0001	0.477
Myocardial infarction	5100 (18.9)	6328 (8.5)	<0.0001	0.305
Obesity	6800 (25.2)	7056 (9.5)	<0.0001	0.424
Chronic kidney disease ≥stage 3	8582 (31.8)	7947 (10.7)	<0.0001	0.534
End‐stage renal disease	5586 (20.7)	5295 (7.1)	<0.0001	0.401
Cardiomegaly	2162 (8.0)	3372 (4.5)	<0.0001	0.143
Pleural effusion	2591 (9.6)	1626 (2.2)	<0.0001	0.319
Ischemic stroke	2294 (8.5)	4983 (6.7)	<0.0001	0.069
Palpitations	1584 (5.9)	5436 (7.3)	<0.0001	0.059
Secondary PAH	251 (0.9)	89 (0.1)	<0.0001	0.111
Angina	677 (2.5)	1485 (2.0)	<0.0001	0.035
Left bundle branch block	1347 (5.0)	2124 (2.9)	<0.0001	0.110
Tachycardia	2688 (10.0)	5526 (7.4)	<0.0001	0.090

Abbreviations: COPD, chronic obstructive pulmonary disease; HFpEF, heart failure with preserved ejection fraction; PAH, pulmonary arterial hypertension; SD, standard deviation; SND, sinus node dysfunction. SMD, standardized mean difference.

### 3.2 Pacemaker Implantation Rate During Follow‐up

**FIGURE 2:**
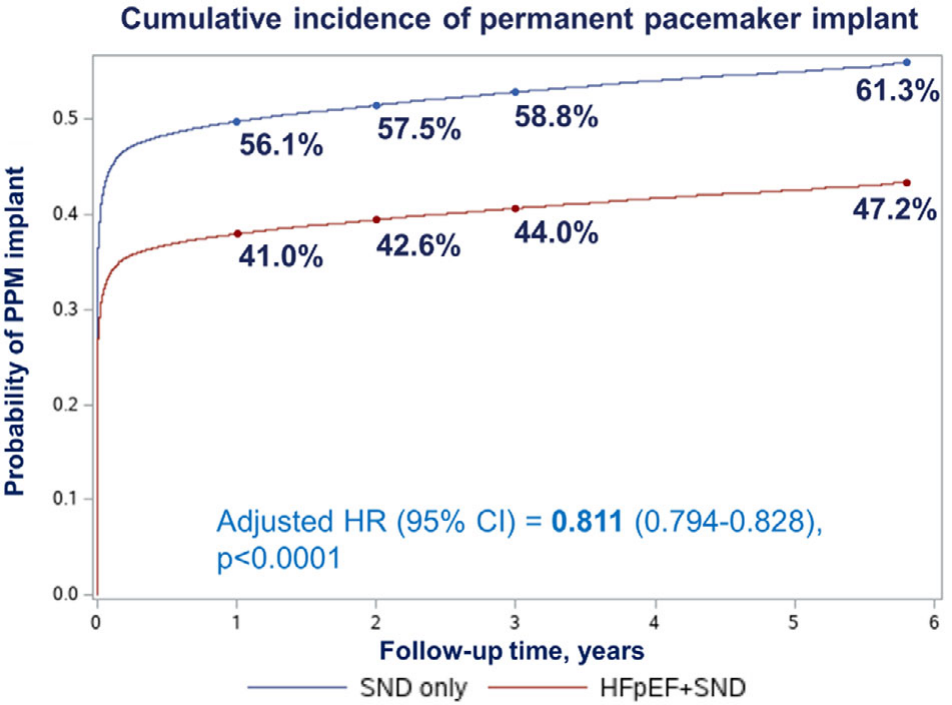
Cumulative incidence of permanent pacemaker implant after SND diagnosis, in patients with vs. without comorbid HFpEF. CI, confidence interval; HFpEF, heart failure with preserved ejection fraction; HR, hazard ratio; PPM, permanent pacemaker, SND, sinus node dysfunction.

**FIGURE 3:**
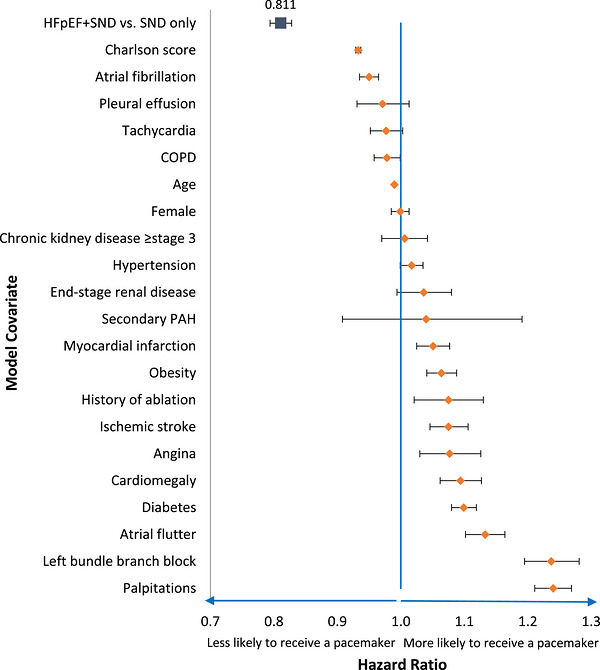
Detailed model results—permanent pacemaker implant post SND diagnosis, in patients with vs. without comorbid HFpEF. *Note*: An increase in Hazard Ratio indicates a higher likelihood of permanent pacemaker implant. COPD, chronic obstructive pulmonary disease; HFpEF, heart failure with preserved ejection fraction; PAH, pulmonary arterial hypertension; SND, sinus node dysfunction.

As shown in Figure 2, the PPM implant rate during follow‐up was markedly lower in the comorbid HFpEF+SND population compared to the SND‐only population (unadjusted HR = 0.694, 95% CI 0.681–0.707, *p* < 0.001; adjusted HR = 0.811, 95% CI 0.794–0.828, *p* < 0.001). At two years of follow‐up, PPM had been implanted in 42.6% of HFpEF+SND versus 57.5% of SND‐only patients, with most implants occurring early in the follow‐up in both cohorts (mean ± SD 38 ± 158 and 40 ± 172 days, respectively) (Table 2). Of the 11,170 HFpEF+SND patients that ultimately received a PPM, 10,232 (91.6%) were transvenous pacemakers, 786 (7.0%) were leadless devices, and 152 (1.4%) were CRT‐Ps; of the 42,753 SND‐only patients with implants, proportions were 41,124 (96.2%) transvenous, 1343 (3.1%) leadless, and 286 (0.7%) CRT‐P. Patients with a history of acute MI, obesity, ischemic stroke, angina, cardiomegaly, diabetes, atrial flutter, LBBB, and palpitations were more likely to be implanted with a PPM (Figure 3).

**TABLE 2:** Characteristics of pacemaker implant.

	HFpEF+SND	SND only
***N***	26,986	74,269
**Time to implant, in days**		
Mean (SD)	38 (158)	40 (172)
Median (IQR)	0 (0–0)	0 (0–2)
**Pacemaker type, *n* (%)**		
CRT‐P	152 (1.4)	286 (0.7)
Leadless	786 (7.0)	1343 (3.1)
Transvenous	10,232 (91.6)	41,124 (96.2)

Abbreviations: CRT‐P, cardiac resynchronization therapy‐pacemaker; HFpEF, heart failure with preserved ejection fraction; IQR, interquartile range; SD, standard deviation; SMD, standard mean difference; SND, sinus node dysfunction.

### 3.3 Clinical Events During Follow‐up

**FIGURE 4:**
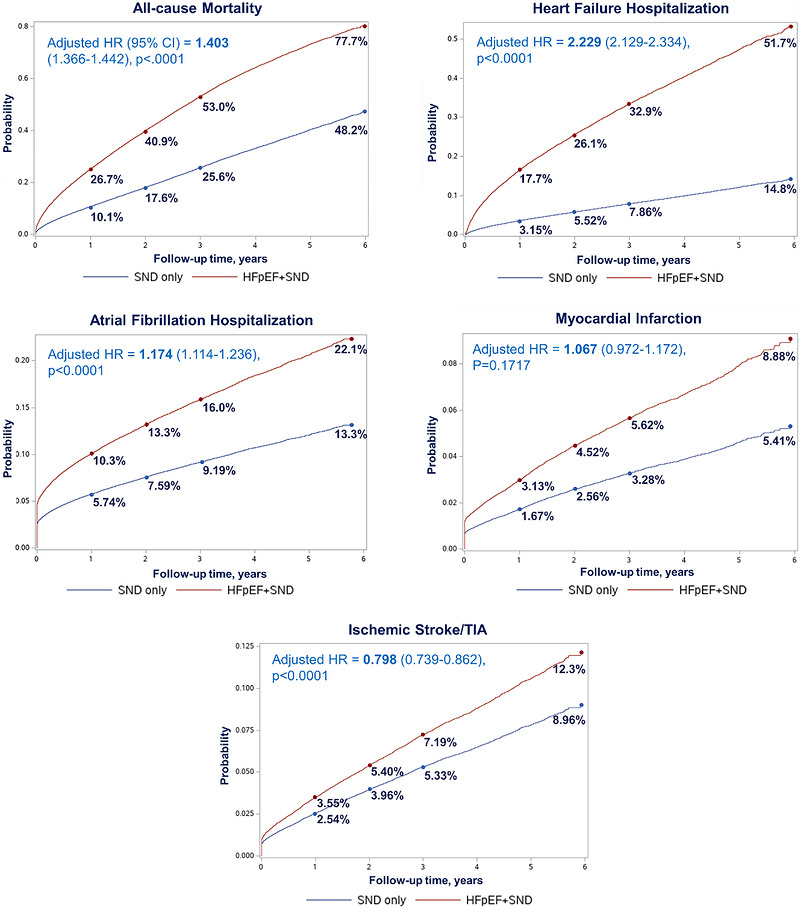
Cumulative incidence of clinical events after SND diagnosis, in patients with vs. without comorbid HFpEF.

Rates of adverse clinical events during follow‐up were significantly higher in the HFpEF+SND population, except for stroke and MI. (Figure 4). After controlling for baseline characteristics, the comorbid HFpEF+SND group had a 40.3% higher all‐cause mortality rate compared to the SND‐only group (unadjusted HR = 2.552, 95% CI 2.497–2.608, *p* < 0.001; adjusted HR = 1.403, 95% CI 1.366–1.442, *p* < 0.001). The rate of HF hospitalization was significantly higher in the comorbid population (unadjusted HR = 4.969, 95% CI 4.794–5.150, *p* < 0.001; adjusted HR = 2.229, 95% CI 2.129–2.334, *p* < 0.001); AF hospitalizations were also more common (unadjusted HR = 1.795, 95% CI 1.722–1.781, *p* < 0.001; adjusted HR = 1.174, 95% CI 1.114–1.236, *p* < 0.001). There was no statistical difference in the adjusted risk of MI between the comorbid HFpEF+SND and the SND‐only populations (unadjusted HR = 1.748, 95% CI 1.627–1.877, *p* < 0.001; adjusted HR = 1.067, 95% CI 0.972–1.172, *p* = 0.1717). After adjustment, the risk of stroke was lower in the HFpEF+SND population (unadjusted HR = 1.375, 95% CI 1.292–1.462, *p *< 0.001; adjusted HR = 0.798, 95% CI 0.739–0.862, *p* < 0.001). Detailed estimates from the models are listed in Appendix Table A3.

### 3.4 Clinical Diagnoses During Follow‐up

**FIGURE 5:**
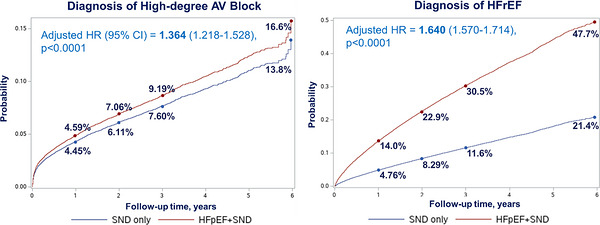
Cumulative incidence of clinical diagnoses after SND diagnosis, in patients with vs. without comorbid HFpEF. AV, atrioventricular; CI, confidence interval; HFpEF, heart failure with preserved ejection fraction; HFrEF, heart failure with reduced ejection fraction; HR, hazard ratio; SND, sinus node dysfunction.

The rate of incident diagnosis of second/third‐degree AV block was higher in the comorbid HFpEF+SND population (adjusted HR = 1.364, 95% CI 1.218–1.528, *p* < 0.001), as shown in Figure 5. The risk of incident diagnosis of heart failure with reduced ejection fraction (HFrEF) was also elevated in comorbid HFpEF+SND patients (adjusted HR = 1.640, 95% CI 1.570–1.714, *p* < 0.001). In the cohort of patients with SND‐only at index, 13.1% of patients went on to be diagnosed with HFpEF at two years of follow‐up (Appendix Table A4). Of these patients, 55.6% had a PPM implantation, with most of them (88.2%) occurring before the HFpEF diagnosis. For the SND‐only patients not diagnosed with HFpEF during follow‐up, the PPM implantation rate at 2 years was 57.7%. Detailed estimates from the models are listed in Appendix Table A3.

### 3.5 Sex Differences in Clinical Outcomes

Hazard ratios for each clinical outcome with an interaction of sex and HFpEF+SND/SND‐only status are shown in Appendix Table A5. When comparing females to males, HFpEF+SND was associated with an increased risk of several outcomes, including AF hospitalization, HFrEF diagnosis, and all‐cause mortality, whereas there was no statistically significant difference in the other outcomes. Additionally, after this stratification, there was no increased risk of AF hospitalization among males with HFpEF+SND versus SND‐only.

## 4 Discussion

This study utilized a large, real‐world US claims database to examine differences in pacemaker utilization and the overall disease burden in SND patients with versus without HFpEF. At the time of SND diagnosis, 25% of patients had comorbid HFpEF; this increased to 36% within two years post‐SND diagnosis. After adjusting for baseline differences, patients with SND and comorbid HFpEF were less likely to receive a PPM implant during follow‐up despite incurring more adverse clinical events (all‐cause mortality, HF hospitalizations, AF hospitalizations) and other significant clinical diagnoses (high‐degree AV block, HFrEF). The increased risk of adverse clinical events are likely related to the greater disease burden in the SND+HFpEF group compared to the SND‐only group, and should not be attributed to a protective effect of pacemaker therapy. Comorbid HFpEF was associated with more MI and ischemic stroke events, but these associations disappeared (in the case of MI) or reversed (in the case of ischemic strokes) after accounting for baseline differences. The reversal in ischemic strokes could be related to differences in baseline AF burden, AF progression, anticoagulation therapy, or left atrial appendage occlusion procedures. When stratifying by sex, the association of HFpEF with mortality, AF hospitalizations, and HFrEF diagnosis was stronger among females than men, and interestingly, the association between HFpEF and AF hospitalization was not seen when examining males alone, indicating a potential impact of sex on the additional comorbidity burden associated with HFpEF. Lastly, among those in the SND‐only cohort, 13.1% went on to be diagnosed with HFpEF within two years of follow‐up. Including the 26.7% of the cohort that had a HFpEF diagnosis on or before their date of SND diagnosis, this brings the cumulative prevalence of HFpEF at two years after SND diagnosis to 36.3%.

### 4.1 HFpEF and SND

The finding that patients with HFpEF and comorbid SND have a higher accrual rate of clinical events than patients with SND alone, even after adjusting for baseline differences, aligns with prior findings that demonstrate a poor prognosis for HFpEF patients and provides validation in a large, real‐world cohort [4, 5, 6]. What is surprising is that more than 10% of the patients with “isolated” SND also received a HFpEF diagnosis in less than two years, which may suggest that the conditions are linked; this accounts for a total incidence of HFpEF among SND patients at 36% at two years post SND diagnosis. In our study, this progression is not linked to the presence (or absence) of pacemaker therapy; future studies in this area should investigate the relationship between pacemaker therapy and progression to HFpEF.

Previous literature shows that advanced age, obesity, and hypertension are among the more predictive conditions of HFpEF [7]. The high prevalence of hypertension (70.3%) in the SND group suggests that many of these patients may have preclinical HFpEF. This notion is also supported by the finding that more than 1 in 3 patients in the SND cohort had baseline AF, which is closely associated with HFpEF [8]. In a commonly used tool for the diagnosis of HFpEF, the presence of AF has the most weight in predicting HFpEF, lending additional support for a link between SND and HFpEF [7]. Our finding that HFpEF patients with SND were less likely to receive PPM therapy could be explained by the common assumption that patients with HFpEF derive a benefit from low heart rates by prolonging diastolic filling time [1]. This rationale explains why beta‐blockers are commonly used in HFpEF despite the lack of supporting evidence from clinical trials [9]. The inclination to maintain lower heart rates in HFpEF, however, is in direct conflict with the less appreciated, but consistent finding that elevating heart rates by pacing can effectively reduce cardiac filling pressures—the main driver of HFpEF. In contrast, low heart rates increase filling pressures, leading to higher atrial and ventricular wall stress, which explains why AF and HFpEF coexist [10, 11, 12, 13]. The realization that low heart rates can have adverse hemodynamic effects in HFpEF led the 2022 AHA/ACC/HFSA heart failure guideline committee to rescind the previous beta‐blocker recommendation for patients with HFpEF [1].

### 4.2 Pacing as a Treatment for HFpEF (and AF)

There is a growing body of evidence that accelerated lower rate pacing may present a treatment opportunity in patients with HFpEF or other cardiomyopathies with restrictive filling, provided pacing is delivered in a manner that supports normal electromechanical activation (i.e. comprehensive conduction system pacing), to minimize the risk of pacing‐induced dyssynchrony. The benefit of accelerated rate pacing on health status, including quality of life, physical activity, and natriuretic peptide levels was demonstrated in the myPACE trial [12]. This study also revealed that the pacing‐mediated reduction in filling pressures can reduce AF burden. The benefits of accelerated pacing were also demonstrated in the related patient group with hypertrophic cardiomyopathy and cardiac amyloidosis [14, 15]. This emerging treatment approach will be further evaluated in the international multicenter ELEVATE‐HFpEF trial, which aims to enroll about 500 HFpEF patients, who will be randomized to a PPM in sensing mode or an accelerated physiologic pacing regimen. Irrespective of the outcomes of this and other ongoing studies such as the PACE HFpEF and FIRE HFpEF trial, this analysis of the Medicare US claims database suggests that the rate of PPM treatment is lower in pacing‐eligible SND patients with comorbid HFpEF versus those with SND without a diagnosis of HFpEF.

### 4.3 Limitations

The accuracy and reliability of administrative claims data to identify all healthcare encounters related to the conditions of interest depends on the existence of specific diagnosis and procedure codes, and the precision and completeness of coding practices, which may vary across providers or institutions. The HFpEF definition is particularly sensitive to miscoding since diagnosis codes don't explicitly identify this condition. Additionally, the lack of clinical data (ejection fraction, QRS duration, etc.) in administrative claims increases the reliance on diagnosis codes and the possibility of misclassification. Cause of death is not available within the Medicare administrative claims dataset, and the study did not include linkage to the national Social Security Administration death master file, hence only all‐cause mortality is reported. Additionally, there may be important underlying patient characteristics not available in claims data that would be unaccounted for in the statistical adjustments for comparisons between the study cohorts. This analysis does not include a comparison of clinical outcomes in patients with versus without PPM treatment, as the possibility of confounding due to selection bias in this real‐world database would limit the reliability of this comparison; this limitation also applies to the progression to HFpEF in the SND‐only cohort, where we can't causally link pacemaker utilization to incidence of HFpEF. Future prospective randomized studies should focus on treatment efficacy in the population with HFpEF.

## 5 Conclusions

In a large contemporary sample of US patients diagnosed with SND at age ≥65 years, over one third developed HFpEF within two years of diagnosis. Compared to those with SND alone, those with SND plus comorbid HFpEF had a lower PPM implant rate while incurring greater rates of all‐cause mortality, HF and AF hospitalizations, and diagnosis of high‐degree AV block and HFrEF. These data suggest that the rate of PPM treatment is lower in pacing‐eligible SND patients with comorbid HFpEF versus those with SND alone. These data provide valuable input into the discussion on pacemaker therapy in HFpEF patients, and further research should examine whether the lower rate of PPM therapy impacts clinical outcomes in these patients. Future work will need to focus on optimizing treatment pathways for HFpEF patients that consider SND and below‐normal heart rates as possible contributors to HFpEF progression, or the benefits of comprehensive conduction system pacing may offer in terms of HFpEF‐modifying effects and the reduction of adverse clinical outcomes, such as AF, that commonly occur in these patients.

## Funding

This study was funded by Medtronic, Inc.

## Conflicts of Interest

Nicole Habel has received research funding and honoraria from Medtronic. Sarah Rosemas, Patrick Zimmerman, and Lucas Higuera are employees with equity interest in Medtronic. Markus Meyer has received research funding from Medtronic and the Engdahl Family Foundation. Markus Meyer and the University of Vermont have licensed patents for the use of pacemakers for the prevention and treatment of atrial fibrillation and heart failure with preserved ejection fraction.

## Supporting information




**Supporting File 1**: pace70172‐sup‐0001‐Appendices.docx
