# Supplementary material for: Real‐World Treatment of Patients With Sinus Node Dysfunction and Heart Failure With Preserved Ejection Fraction
Source: Pacing Clin Electrophysiol. 2026 Feb 26;49(8):1015–22. doi: 10.1111/pace.70172 (PMC13456363; doi:10.1111/pace.70172)
Supplement: Supplementary file 1 [file PACE-49-1015-s001.docx]

**Appendices**

**Real-world Treatment of Patients with Sinus Node Dysfunction and Heart Failure with Preserved Ejection Fraction**

Appendix Table A1. Diagnosis codes

| **Event** | **ICD-10-CM codes** |
| --- | --- |
| **AV Block 2^nd^/3^rd^ degree** | I44.1, I44.2, I44.30, I44.39 |
| **Sinus node dysfunction** | I49.5 |
| **Systolic HF^1^** | I50.2, I50.20, I50.21, I50.22, I50.23, I50.40, I50.41, I50.42, I50.43 |
| **Diastolic or Combined HF^1^** | I50.3, I50.30, I50.31, I50.32, I50.33 |
| **Hypertension** | I10, I11.0, I11.9, I12.0, I12.9, I13.0, I13.10, I13.11, I13.2, I15.0, I15.1, I15.2, I15.8, I15.9, I16.0, I16.1, I16.9, I97.3 |
| **Atrial fibrillation^1^** | I48.0, I48.1, I48.11, I48.19, I48.2, I48.20, I48.21, or I48.91 |
| **Atrial Flutter** | I48.3, I48.4, I48.92 |
| **COPD** | J43.0, J43.1, J43.2, J43.8, J43.9, J44.0, J44.1, J44.81, J44.89, J44.9, J47.0, J47.1, J47.9, J60 |
| **Diabetes** | E08.x, E09.x, E10.x, E11.x, E13.x |
| **Myocardial Infarction^1^** | I21.01, I21.02, I21.09, I21.11, I21.19, I21.21, I21.29, I21.3, I21.4, I21.9, I21.A1, I21.A9, I21.B, I22.0, I22.1, I22.2, I22.8, I22.9, I25.2 |
| **Ischemic stroke/Transient ischemic attack^1^** | I63.00, I63.011, I63.012, I63.013, I63.019, I63.02, I63.031, I63.032, I63.033, I63.039, I63.09, I63.10, I63.111, I63.112, I63.113, I63.119, I63.12, I63.131, I63.132, I63.133, I63.139, I63.19, I63.20, I63.211, I63.212, I63.213, I63.219, I63.22, I63.231, I63.232, I63.233, I63.239, I63.29, I63.30, I63.311, I63.312, I63.313, I63.319, I63.321, I63.322, I63.323, I63.329, I63.331, I63.332, I63.333, I63.339, I63.341, I63.342, I63.343, I63.349, I63.39, I63.40, I63.411, I63.412, I63.413, I63.419, I63.421, I63.422, I63.423, I63.429, I63.431, I63.432, I63.433, I63.439, I63.441, I63.442, I63.443, I63.449, I63.49, I63.50, I63.511, I63.512, I63.513, I63.519, I63.521, I63.522, I63.523, I63.529, I63.531, I63.532, I63.533, I63.539, I63.541, I63.542, I63.543, I63.549, I63.59, I63.6, I63.81, I63.89, I63.9, G45.0, G45.1, G45.2, G45.3, G45.4, G45.8, G45.9 |
| **Obesity** | E66.01, E66.09, E66.1, E66.2, E66.8, E66.9 |
| **CKD ≥3^rd^ stage** | N18.1, N18.2, N18.30, N18.31, N18.32, N18.4, N18.5, N18.9 |
| **ESRD** | N18.9 |
| **Pleural effusion** | J90, J91.0, J91.8 |
| **Cardiomegaly** | I51.7 |
| **Palpitations** | R00.2 |
| **Secondary pulmonary arterial hypertension** | I27.21 |
| **Angina** | I20.0, I20.1, I20.8, I20.89, I20.9, I23.7 |
| **Left bundle branch block** | I44.7, I45.2, I45.3, I44.60 |
| **Tachycardia** | R00.0, I47.1, I49.2, I47.0, I47.20, I47.21, I47.29, I47.9 |

1 When used to identify outcomes (HF hospitalization, AF hospitalization, ischemic stroke/TIA, myocardial infarction), only the admitting or first diagnosis position of inpatient claims is used.

Abbreviations: AV, atrioventricular; CKD, chronic kidney disease; COPD, chronic obstructive pulmonary disease; HF, heart failure.

Appendix Table A2. Procedure codes

| **Event** | **CPT Codes** | **ICD-10-PCS codes** | **ICD-10-CM diagnosis codes** | |
| --- | --- | --- | --- | --- |
| **AF Ablation** | 93653, 93656, 93657 | 02583ZZ + Diagnosis^1^ | I48.0, I48.1, I48.11, I48.19, I48.2, I48.20, I48.21, I48.91 | |
| **AV node ablation** | 93650 | 02583ZZ + Diagnosis^1^ | I48.0, I48.1, I48.11, I48.19, I48.2, I48.20, I48.21, I48.3, I48.4, I48.91 or I48.92 | |
| **Septal Puncture (indicates not AV node ablation)** | N/A | 4A023N7 | N/A | |
| **Leadless IPG** | 0387T, 33274 | 02HK3NZ | N/A | |
| **Single-chamber IPG** | 33206 without 33225 | 0JH604Z or 0JH605Z | N/A | |
|  | 33207 without 33225 |  |  |  |
| **Dual-chamber IPG** | 33208 without 33225 | 0JH606Z | N/A | |
| **Pacemaker system replacement** | 33227 or 33228 | 0JH604Z or 0JH605Z or 0JH606Z, plus 0JPT0PZ |  | |
| **Coronary lead or left ventricular lead** | 33225 | 02H43JZ or 02HL3JZ | N/A | |
| **CRT-P implant** | 33208 and 33225 | 0JH607Z plus (02H43JZ or 02HL3JZ) | N/A | |
| **CRT-P replacement** | 33229 | 0JH607Z plus 0JPT0PZ | N/A | |
| **ICD implant** | 33249, 33270 (and no 33225) | 0JH608Z | N/A | |
| **ICD replacement** | 33262, 33263 | 0JH608Z plus 0JPT0PZ | N/A | |
| **CRT-D implant** | 33249 plus 33225 | 0JH609Z | N/A | |
| **CRT-D replacement** | 33264 | 0JH609Z plus 0JPT0PZ | N/A | |
| **Remove CIED** | N/A | 0JPT0PZ | N/A | |
| **Loop diuretic infusion** | J3265, S0171, J1940 | N/A | N/A | |
| Abbreviations: CIED, cardiovascular implantable electronic device; CRT-P, chronic resynchronization therapy-pacemaker; ICD, implantable cardioverter-defibrillator; IPG, implantable pulse generator. ^1^Diagnosis code in first or admitting position in same encounter as procedure code. | | | |  |

Appendix Table A3. Detailed multivariable model results – Cumulative incidence of clinical events and clinical diagnoses post SND diagnosis, in patients with vs. without comorbid HFpEF

|  | **Ischemic Stroke/TIA** | | **Myocardial Infarction** | | **AF Hospitalization** | | **HF Hospitalization** | | **Diagnosis of HFrEF** | | **Diagnosis of 2^nd^/3^rd^ Degree AV Block** | | **All-cause mortality** | |
| --- | --- | --- | --- | --- | --- | --- | --- | --- | --- | --- | --- | --- | --- | --- |
| **Model covariate** | **HR** | **95% CI** | **HR** | **95% CI** | **HR** | **95% CI** | **HR** | **95% CI** | **HR** | **95% CI** | **HR** | **95% CI** | **HR** | **95% CI** |
| **HFpEF+SND vs. SND only** | **0.798** | **(0.739-0.862)** | **1.067** | **(0.972-1.172)** | **1.174** | **(1.114-1.236)** | **2.229** | **(2.129-2.334)** | **1.64** | **(1.570-1.714)** | **1.364** | **(1.218-1.528)** | **1.403** | **(1.366-1.442)** |
| **Age** | 1.021 | (1.018-1.025) | 1.002 | (0.998-1.006) | 0.988 | (0.986-0.991) | 1.022 | (1.019-1.024) | 1.008 | (1.006-1.010) | 1.006 | (1.001-1.012) | 1.069 | (1.068-1.071) |
| **Charlson score** | 1.105 | (1.092-1.118) | 1.096 | (1.080-1.111) | 1.016 | (1.005-1.026) | 1.058 | (1.049-1.067) | 1.003 | (0.994-1.011) | 1.01 | (0.988-1.032) | 1.164 | (1.158-1.171) |
| **Female** | 1.161 | (1.096-1.230) | 0.936 | (0.872-1.004) | 1.463 | (1.404-1.525) | 1.218 | (1.173-1.266) | 0.809 | (0.781-0.837) | 0.759 | (0.695-0.829) | 0.862 | (0.843-0.881) |
| **Diabetes** | 1.031 | (0.962-1.106) | 1.248 | (1.148-1.356) | 0.907 | (0.863-0.953) | 1.208 | (1.156-1.263) | 1.227 | (1.177-1.278) | 1.048 | (0.941-1.166) | 0.941 | (0.916-0.968) |
| **Hypertension** | 1.044 | (0.968-1.126) | 1.136 | (1.030-1.252) | 0.925 | (0.876-0.977) | 1.129 | (1.065-1.196) | 1.12 | (1.066-1.178) | 1.273 | (1.128-1.438) | 0.967 | (0.939-0.996) |
| **Atrial fibrillation** | 0.928 | (0.872-0.987) | 0.716 | (0.663-0.774) | 2.947 | (2.809-3.092) | 1.397 | (1.340-1.458) | 1.383 | (1.331-1.438) | 0.861 | (0.779-0.951) | 1.076 | (1.051-1.102) |
| **Atrial flutter** | 0.866 | (0.770-0.974) | 0.9 | (0.780-1.039) | 1.136 | (1.066-1.210) | 1.061 | (0.997-1.129) | 1.01 | (0.950-1.073) | 1.41 | (1.208-1.645) | 0.955 | (0.916-0.995) |
| **COPD** | 0.921 | (0.852-0.996) | 0.924 | (0.842-1.015) | 0.947 | (0.898-0.998) | 1.208 | (1.156-1.263) | 1.115 | (1.067-1.165) | 0.937 | (0.759-1.005) | 1.237 | (1.203-1.273) |
| **Myocardial infarction** | 1.039 | (0.954-1.133) | 2.277 | (2.086-2.484) | 1.022 | (0.961-1.087) | 1.059 | (1.005-1.115) | 1.364 | (1.300-1.431) | 0.873 | (0.759-1.005) | 0.949 | (0.917-0.982) |
| **Obesity** | 0.941 | (0.860-1.030) | 0.845 | (0.760-0.939) | 0.967 | (0.898-0.998) | 1.277 | (1.216-1.341) | 1.136 | (1.083-1.192) | 1.137 | (1.000-1.292) | 0.968 | (0.935-1.002) |
| **Chronic kidney disease ≥stage 3** | 0.721 | (.629-.827) | 0.91 | (0.786-1.054) | 0.819 | (0.746-0.898) | 1.074 | (1.000-1.153) | 1.028 | (0.955-1.106) | 1.05 | (0.877-1.258) | 1.047 | (1.000-1.096) |
| **End-stage renal disease** | 1.097 | (0.942-1.279) | 0.92 | (0.780-1.085) | 0.946 | (0.851-1.052) | 1.089 | (1.009-1.176) | 1.104 | (1.019-1.196) | 0.854 | (0.692-1.054) | 0.96 | (0.912-1.011) |
| **Ischemic stroke** | 2.050 | (1.890-2.223) | 0.936 | (0.823-1.064) | 0.812 | (0.750-0.880) | 0.8 | (0.745-0.859) | 0.892 | (0.835-0.953) | 1.026 | (0.870-1.210) | 0.923 | (0.886-0.961) |
| **Pleural effusion** | 0.762 | (0.653-0.888) | 0.713 | (0.593-0.857) | 0.819 | (0.743-0.903) | 1.111 | (1.033-1.195) | 1.057 | (0.982-1.139) | 0.978 | (0.792-1.208) | 1.39 | (1.323-1.460) |
| **Cardiomegaly** | 0.949 | (0.837-1.076) | 0.997 | (0.858-1.159) | 1.002 | (0.925-1.085) | 1.268 | (1.185-1.356) | 1.379 | (1.296-1.468) | 1.103 | (0.921-1.320) | 0.922 | (0.881-0.965) |
| **Palpitations** | 0.876 | (0.775-0.992) | 0.975 | (0.841-1.130) | 1.245 | (1.165-1.330) | 0.864 | (0.799-0.934) | 0.946 | (0.882-1.015) | 0.891 | (0.740-1.074) | 0.607 | (0.575-0.640) |
| **Secondary pulmonary arterial hypertension** | 0.544 | (0.283-1.046) | 0.39 | (0.162-0.942) | 0.694 | (0.490-0.983) | 1.194 | (0.953-1.497) | 1.082 | (0.851-1.376) | 0.862 | (0.411-1.807) | 1.211 | (1.017-1.441) |
| **Angina** | 1.234 | (1.035-1.471) | 1.327 | (1.094-1.609) | 0.998 | (0.877-1.136) | 0.997 | (0.885-1.123) | 1.176 | (1.060-1.303) | 0.895 | (0.657-1.220) | 0.799 | (0.738-0.866) |
| **Left bundle branch block** | 0.913 | (0.780-1.069) | 0.978 | (0.817-1.170) | 0.915 | (0.823-1.018) | 1.181 | (1.087-1.282) | 1.431 | (1.327-1.542) | 1.143 | (0.922-1.417) | 0.831 | (0.783-0.883) |
| **Tachycardia** | 0.949 | (0.852-1.058) | 0.906 | (0.793-1.035) | 1.173 | (1.101-1.250) | 0.913 | (0.855-0.975) | 0.935 | (0.879-0.996) | 1.008 | (0.859-1.182) | 0.959 | (0.919-1.000) |
| **History of ablation** | 0.942 | (0.713-1.243) | 0.761 | (0.531-1.090) | 1.369 | (1.223-1.532) | 1.056 | (0.918-1.215) | 1.069 | (0.939-1.216) | 0.819 | (0.564-1.188) | 0.634 | (0.563-0.714) |

Abbreviations: AF, atrial fibrillation; AV, atrioventricular; CI, confidence interval; COPD, chronic pulmonary obstructive disease; HF heart failure; HFrEF, heart failure with reduced ejection fraction; HR, hazard ratio.

Appendix Table A4. Cumulative incidence of HFpEF diagnosis in SND cohort

Abbreviations: HFpEF, heart failure with preserved ejection fraction; SND, sinus node dysfunction.

Appendix Table A5. Statistical interaction of clinical event rates and sex

|  | **Overall**  **HR (95% CI)** | **Females only**  **HR (95% CI)** | **Males only**  **HR (95% CI)** | **Interaction  p-value** |
| --- | --- | --- | --- | --- |
| **Pacemaker implant** | 0.811 (0.794-0.828) | 0.822 (0.802-0.843) | 0.794 (0.769-0.820) | 0.0737 |
| **Clinical events:** |  |  |  |  |
| Stroke | 0.798 (0.739-0.862) | 0.826 (0.755-0.903) | 0.75 (0.667-0.844) | 0.1622 |
| Myocardial Infarction | 1.067 (0.972-1.172) | 1.106 (0.987-1.239) | 1.019 (0.897-1.158) | 0.2887 |
| AF hospitalization | 1.174 (1.114-1.236) | 1.238 (1.167-1.313) | 1.041 (0.959-1.13) | **0.0002** |
| HF hospitalization | 2.229 (2.129-2.334) | 2.192 (2.076-2.313) | 2.292 (2.146-2.447) | 0.2466 |
| All-cause mortality | 1.403 (1.366-1.442) | 1.436 (1.390-1.484) | 1.359 (1.307-1.413) | **0.0192** |
| **Clinical diagnoses:** |  |  |  |  |
| HFrEF | 1.64 (1.570-1.714) | 1.758 (1.665-1.856) | 1.51 (1.424-1.601) | **<.0001** |
| AV Block | 1.364 (1.218-1.528) | 1.409 (1.223-1.624) | 1.313 (1.128-1.528) | 0.4531 |

Abbreviations: AF, atrial fibrillation; AV, atrioventricular; HF, heart failure; HFrEF, heart failure with reduced ejection fraction; HR, hazard ratio.
